# 

**Published:** 1911-03-18

**Authors:** 


### THE PHYSIOLOGICAL FEEDING OF INFANTS.

THIRD EDITION.

**A Complete Handbook of the Principles and Practice of Infant Feeding.**

**By ERIC PRITCHARD, M.A., M.D. (Oxon.),** Assistant Physician to the Queen's Hospital for Children Physician to Out-Patients, City of London Hospital for Diseases of the Chest, &c.

"This was a good book in its first edition, it is a much better book now."—*Lancet.* Price **7s. 6d.** net.

### Publications.

**By A. H. TUBBY, M.S.**

### DEFORMITIES.

Six hundred pages, 300 Illustrations. Price 17s.
"Standard work on the subject in the English language."
*British Medical Journal.*

### SURGERY OF PARALYSES.

By A. H. TUBBY, M.S.Lond., F.R.C.S.Eng., and ROBERT JONES, F.R.C.S.E.
Illustrated by 93 figures and 68 cases. Pp. 301. 10s. net.
"Can confidently recommend it as a practical summary of the most modern views."—*Edinburgh Medical Journal.*
"Indicates original and successful methods of treatment."
*American Journal of Orthopœdic Surgery.*

London: Macmillan & Co., Ltd. New York: The Macmillan Co.

### THERAPEUTICS OF THE CIRCULATION

By Sir LAUDER BRUNTON, Bart., M.D., D.Sc., F.R.C.P., &c., Consulting Physician to St. Bartholomew's Hospital.
Published under the Auspices of the University of London.
With numerous Diagrams. Medium 8vo. 7s. 6d. net.

JOHN MURRAY, Albemarle Street, W.

### "THE PRESCRIBER." Vol. IV., 1910.

A valuable Book of Reference for the General Practitioner.
Contains all that has happened during the year in connection with Therapeutics and New Remedies, with a **very full Index.**
*THE HOSPITAL* says:—"There is not a page of useless knowledge in the whole volume; there are many pages of sterling worth, which give the book a permanent value."

Cloth, 7s. 6d. net; Half-Leather, 9s. 6d. net.
*Post free in United Kingdom. Abroad 6d. extra.*
**"The Prescriber" Offices, 137 George St., Edinburgh**

**By Sir WILLIAM BENNETT, K.C.V.O., F.R.C.S.**

**RECENTLY PUBLISHED. Crown 8vo. 5s. net.**

### INJURIES AND DISEASES OF THE KNEE JOINT.

With 34 Illustrations.

NISBET & CO.

**Also, 8vo. 6s. FOURTH EDITION.**

### MASSAGE AND MOVEMENTS IN RECENT FRACTURES;

**Sprains and their Consequences; Rigidity of the Spine and the Management of Stiff Joints.**
With 23 Illustrations.
LONGMANS, GREEN & CO.

Just Issued. Ninth Edition, Rewritten and Enlarged, 9s. net.

### PHARMACY, MATERIA MEDICA & THERAPEUTICS.

Being a Commentary on the B.P. and its Drugs, with their Preparations, and on all the new Non-official Remedies in use, with Chapters on the different processes used in Dispensing, Prescription-writing, Latin Glossary, &c. By **Sir W. WHITLA, M.D., LL.D.,** Professor, Materia Medica, Queen's University, Belfast, and Senior Physician, Royal Victoria Hospital.

By the same Author. New Work. 1,900 pages. Crown 8vo. 25s. net.

### PRACTICE OF MEDICINE.

Containing in two Handy Volumes a complete and independent Article on the Etiology, Symptoms, and Diagnosis of every Medical Disease; arranged in Dictionary Form for Practitioners and Students.

London: Baillière, Tindall & Cox, 8 Henrietta Street, W.C.

FIFTH EDITION. With 50 Illustrations. 5/- net.

### THE SCHOTT METHODS OF THE TREATMENT OF CHRONIC DISEASES OF THE HEART.

With an Account of the Nauheim Baths and of their Therapeutic Exercises
By W. BEZLY THORNE, M.D., M.R.C.P.
London: J. & A. Churchill, 7 Great Marlborough Street.

Lieutenant-Colonel Alfred William Frederick Street, D.S.O., of Norsey Manor, Billericay, Essex, late of the Indian Medical Service, who saw service in the Burmese Expedition, 1886-87, and who died on January 30, aged forty-eight, has left estate valued at £2463 gross, with net personalty £2375.

### SMALLPOX IN LONDON.

From a return issued by the Statistical Department of the Metropolitan Asylums Board it appears that up to the week ending March 11 there were 52 cases of smallpox notified in London. Of these patients 39 were said to have been vaccinated in infancy, and most of them bore vaccination scars. None of them had ever been re-vaccinated, and one out of the 39 had died. Of the 13 other patients, none had ever been vaccinated; five of this group have died. Comment is unnecessary on these figures. But it is high time that a Ministry of Public Health should be created, free from the degrading association with party politics; for the President of the Local Government Board declines to issue instructions recommending vaccination, owing to the displeasure which such an action would cause among influential sections of his party, and although he himself is quite well aware of the value of vaccination as a prophylactic against smallpox.

### BOOKS RECEIVED.

Food and Cookery Publishing Co.
"Cookery Annual," 1911. By C. Hermann Senn.

Lippincott & Co.
"International Clinics."
"Fever Nursing." By J. C. Wilson, A.M., M.D.

The Scientific Press.
"Massage in Practice." By Miss M. Atkey.
"Aids to Midwifery." By Rebecca Emily Rémy.

### MEDICAL APPOINTMENTS, ETC.

Dr. Sinclair White, M.D., M.Ch. (R.U.I.), F.R.C.S., has been appointed to the Professorship of Surgery in Sheffield University, in succession to Mr R. J. Pye-Smith, resigned.

Hubert Pinto-Leite, B.A. Cantab., M.R.C.S. Eng., L.R.C.P., Lond., has been appointed an anæsthetist for one year to the Hospital for Women, Soho Square, W., in the place of Dr. Maynard Horne, who has been granted absence on leave.

Mr. H. H. Rayner, M.B., F.R.C.S. Eng., has been appointed a visiting surgeon to the Manchester Children's Hospital *vice* Mr. E. D. Telford, F.R.C.S., resigned.

Mr. Gordon J. Lane, M.D., and Mr. E. Challons, M.D., have been appointed assistant hon. physicians, and Mr. H. Tyrrell Gray, F.R.C.S., Mr. Alf. J. Couzens, F.R.C.S., and Mr. Percival P. Cole, F.R.C.S., honorary surgeons at West Ham and Eastern General Hospital.

### OBITUARY.

The death is announced at Alford, Lincolnshire, of Mr. George Bosson, aged sixty-six years. Mr. Bosson took his M.R.C.S. in 1872, becoming in the same year a licentiate of the Society of Apothecaries.

Captain W. Harold Gillatt, R.A.M.C., has died at Cairo. Captain Gillatt studied medicine at the University of Glasgow, where he graduated M.B., B.S., so recently as 1905, subsequently joining the Army Medical Service.

MR. GEORGE SPICER, whose death at his home at Enfield has just occurred, was a good friend to the local cottage hospital. One of those busy men who seem able to find time for everything, Mr. Spicer never allowed his educational, magisterial, political, or fishmonger's interests to interfere with his institutional work. This was the real work of a hospital treasurer, and is all the more praiseworthy as coming from a man more accustomed to the relatively easy work of writing cheques than to the very laborious one of collecting them. The Enfield Cottage Hospital will miss his influence and the pains which he took to secure the annual solvency of his pet local institution. Mr. Spicer was only fifty-six years of age.

The Manchester Children's Hospital has received a legacy of £1,000 from the late President, Sir William Agnew, Bart., and also a legacy of £900 from Mrs. Helen Crowther, and an intimation of a legacy from Miss Sarah Hempson, of Radcliffe, of £1,000.

### The Hospital World.

### Gifts and Bequests.

Mr. Jos. Thomas has left £1,000 to the Pembrokeshire Infirmary.

Mrs. Charlotte Louisa Deacon, of Clifton Park, Clifton, who left estate of the gross value of £46,635, has bequeathed £500 to the Bath General Hospital.

Mr. Frank Gott has remitted the rent of Armley House (the Hospital for Consumptives), Leeds, as a Coronation year gift. This is equivalent to a contribution of £150.

The Provincial Cinematograph Theatres, Limited, kindly having placed the Picture House at Leicester at the disposal of the Leicester Infirmary for a benefit, £75 was thereby raised.

Mr. Samuel Watton Smith, of Edgbaston, Birmingham, chairman of Messrs. J. Brockhouse and Co. (Ltd.), spring manufacturers, has left £100 to the Queen's Hospital, Birmingham, and £100 to the Birmingham and Midland Eye Hospital.

Mr. Samuel Allcock, head of the firm of Messrs. S. Allcock and Co. (Limited), has left £100 each to the Birmingham Hospital for Women, the General Hospital, Birmingham, the Queen's Hospital, Birmingham, the Smallwood Hospital, Redditch, the Ear and Throat Hospital, Birmingham, the Eye Hospital, Birmingham, and the Blackwell Sanatorium.

### Jottings.

The Queen has sent four book rests to the British Home and Hospital for Incurables, Streatham.

The Japanese Ambassador, accompanied by Admiral Sir John Durnford, visited the Dreadnought Seamen's Hospital last Saturday.

Chelsea Hospital for Women.—Annual meeting of Governors on March 30, 4 p.m. Viscount Castlereagh, M.P., M.V.O., President, in the chair.

The 61st annual general meeting of the London Homœopathic Hospital took place in the Board Room of the Hospital on Friday, March 17. John Pakenham Stilwell, Esq., J.P., Chairman of the Board of Management, presided. The new Sir Henry Tyler wing extension was open for inspection by the governors, donors and subscribers from 3.30 to 5.30 o'clock.

Princess Victoria of Schleswig-Holstein, lady president of the League of Mercy for Berkshire, Buckinghamshire, Hampshire, and Oxfordshire, presided at a meeting held, at the invitation of Mrs. Murdoch, at 51 Rutland Gate, last Tuesday. Lady Portal and the Countess of Malmesbury (hon. secretaries) represented Hampshire, Mrs. Murdoch Berkshire, Lady Clayton (hon. secretary) Buckinghamshire, and Viscountess Dillon (hon. secretary) Oxfordshire.

On Tuesday last Sir George White, President of the Bristol Royal Infirmary, laid the foundation-stone of the surgical department which is to be built as a memorial to King Edward. This building is only the first instalment of a larger work which will cost £70,000, of which £35,000 has been raised. No appeal will be made until the King Edward memorial statue scheme is finished.

At the annual meeting of the Court of the Royal Waterloo Hospital for Children and Women, held last Tuesday at the Mansion House, the Lord Mayor presided, and reminded the meeting that the hospital was the outcome of a meeting held in the City as far back as April 1816, when the Lord Mayor of the day was appointed President.

### COMING EVENTS OF THE WEEK.

[Communications for this column must be sent to 29 Southampton Street, Strand, before noon on Wednesday.]

Monday, March 20 : Princess Christian will open at 12 o'clock the sale of work to be given by the Hon. Mrs. Charles Lawrence at 23 Eaton Square, in aid of the Ladies' Association of the Royal Hospital for Incurables, Putney Heath.

Tuesday, March 21 : Simple Life and Healthy Food : Annual Conference and Exhibition opens, Caxton Hall (four days).

League of Mercy, North Kensington District : Lady Furley " At Home," at 14 Evelyn Gardens, at 5 p.m. ; to meet Her Highness Princess Marie Louise of Schleswig-Holstein.

Annual meeting of the Poplar Hospital for Accidents at the Hospital at 4 p.m.

Wednesday, March 22 : Earl Waldegrave will preside at the annual general meeting of subscribers to the Florence Nightingale Hospital (formerly known as the Hospital for Invalid Gentlewomen), at 3 p.m.

Annual general meeting of the subscribers of the Manchester Children's Hospital will be held at the Godfrey Ermen Memorial Dispensary, Gartside Street, Manchester. The Right Hon. the Lord Mayor of Manchester will preside.

Thursday, March 23 : The Right Hon. Lord Alverstone, G.C.M.G., Lord Chief Justice of England, will preside at the annual general meeting of Governors of the Royal National Hospital for Consumption, Ventnor, Isle of Wight, to be held at the London Offices, 18 Buckingham Street, Strand, at 4.30 p.m.

Annual meeting of the Child-Study Society will be held at the Royal Sanitary Institute, 90 Buckingham Palace Road, at 6.30.

City of London Hospital for Diseases of the Chest, Victoria Park : Annual meeting of Governors, Sir Edward Sassoon, M.P., presiding, Cannon Street, Hotel, 3 o'clock.

Invalid Children's Aid Society : Conference, 12 Portland Street, 3.30.

League of Mercy, South Paddington District : A concert will be held at St. Michael's Schools, Star Street, W., at 8.30 p.m.

Friday, March 24 : Belgrave Hospital for Children : Annual "Pound Day." Lady Plymouth opens the proceedings, 2.30.

Tuesday, April 4 : 12 o'clock. Princess Christian will take the chair at the annual meeting of the Ladies' Association of the Hospital for Women, Soho Square, at 31 Great Cumberland Place.

---

According to *Indian Public Health* the opium habit in Burmah seems to have been checked largely owing to the exertions of the Excise Department, but simultaneously there has been a very serious development of the far worse habit of cocaine taking. It is easy to correlate the two events as cause and effect, and this has been done by several of the officers reporting, but in the absence of proof the Commissioner rightly leaves this matter open, and contents himself with showing how rapidly the habit is spreading, how much easier cocaine is to smuggle than opium, and how urgently laws are needed to prevent the evil. It is important that Government should realise the danger of establishing the cocaine habit as well as the part that British firms are taking in implanting it.

### BUILDINGS CONTEMPLATED AND IN PROGRESS.

Billericay.—Mr. Hugo R. Bird is architect for the reconstruction of the District Hospital at Billericay, and the plans were discussed by the Brentwood Urban District Council at their last meeting, the further detail being referred to the General Purposes Committee.

Chichester.—In our issue of February 25 it was reported that comprehensive extensions costing close on £14,000 are anticipated to Chichester Infirmary, for which Mr. Charles W. Ball of Southsea is architect. This sum is now practically all subscribed or promised, and it is proposed to begin building operations forthwith.

Eastleigh and Bishopstoke.—The Urban District Council have approved the amended plans of the Isolation Hospital, and directed that these should be forwarded to the Local Government Board for preliminary approval.

London.—For the new King's College Hospital at Denmark Hill, S.E., for which W. A. Pite, Esq., F.R.I.B.A., is architect, about £200,000 has been subscribed, and a further £150,000 is necessary to carry on the work to its opening stage.

St. Austell.—In reply to the invitation to architects for plans referred to in our issue of January 28 last, seventeen sets of drawings have been submitted to the assessor, Mr. Thornley, of Messrs Thornley and Nooke, Plymouth.

Wemyss.—At a special meeting of Wemyss Parish Council, Bailie Rose, chairman, submitted sketch plans showing alterations and additions to Thornton Poorhouse. The scheme, which is estimated to cost close on £10,000, provides a new hospital and administration buildings.

---

### APPOINTMENTS VACANT.

Full particulars of these vacancies can be obtained on reference to our advertisement columns :—

Leicester Infirmary.—Assistant House Surgeon.

Macclesfield General Infirmary.—Junior House Surgeon.

Royal Free Hospital.—Secretary.

Seamen's Hospital Society.—Two House Physicians, Two House Surgeons.

Tunbridge Wells General Hospital.—House Physician.

West Bromwich District Hospital.—Senior House Surgeon.

Wolverhampton General Hospital.—House Surgeon.

---

Mrs. Mary Helen Christie, of Ribsden, Windlesham, Surrey, who left estate valued at £96,205 gross, has bequeathed £5,000 for the erection of a hospital in India to the Zenana Bible and Medical Mission or Indian Female Normal School, and Instruction Society, Adelphi Terrace; £4,000 to the Northern Counties Hospital for Incurables, for the provision of five pensions of at least £20; £4,000 to the Victoria Dental Hospital, Manchester, of which £1,000 is to be for the Denture Fund; £4,000 to the Manchester Northern Hospital (formerly Clinical Hospital) for Women and Children, Manchester; £4,000 to the Christie Hospital (formerly called the Cancer Pavilion and Home Hospital), Manchester; £3,200 to the Royal Medical College, Epsom, for the provision of two pensions of at least £40 each for medical men; £2,000 to the Manchester and Salford Sick Poor and Private Nursing Institute, for the district nursing branch; £1,000 to the St. Mary's Hospital, Manchester; and £500 to the Infants' Hospital, Vincent Square, Westminster.

**The FUTURE of the HOSPITALS**

*MEDICAL RELIEF FOR ALL ACCORDING TO MEANS.*

A Scheme of Co-operation between the PATIENTS, the HOSPITALS, and the STATE.

Price 2d. By Sir HENRY BURDETT, K.C.B., K.C.V.O. Price 2d.

THE SCIENTIFIC PRESS, Ltd., 28 & 29 Southampton Street, Strand, London, W.C.; and at all Bookstalls.

### The Scientific Press Publications

### *INSTITUTIONAL WORKS.*

### HOSPITALS AND ASYLUMS OF THE WORLD.

By SIR HENRY BURDETT, K.C.B., K.C.V.O.

In Four Volumes, with Separate Portfolio containing several hundred Plans.

Vols. I. and II. (ASYLUMS) ... Price £6 15s.

Vols. III. and IV. (HOSPITALS) and the Portfolio of Plans ... Price £9

The Portfolio of Plans (20 in. × 14 in., drawn to uniform scale), Price £4 14s. 6

The Complete Work ... ... £12 12s.

This magnificent work forms a Complete Survey of the Origin, History, Construction, Administration and Management of the Hospitals and Asylums of the World, with detailed information concerning the principal Institutions.

*Only a few copies remain unsold.*

### LEDGERS, ACCOUNT BOOKS, STOCK BOOKS of all kinds for large and small Institutions.

These are supplied already ruled and printed, or in accordance with the special requirements of institution managers.

### ANNUAL REPORTS AND PROSPECTUSES.

The Scientific Press are prepared to undertake the production of the Annual Reports and Prospectuses of every class of Institution, and to supply printed or typewritten copies of testimonials for the officials. Professional Cards.

### CHARTS and CHART CASES.

Temperature Charts—

| | | |
|---|---|---|
| Morning and Evening | 12 ... | 6d. |
| Four-Hour Charts ... | 25 ... | 1s. |
| Maternity Charts ... | 1,000 ... | 23s. |

### CASE PAPERS

For Day and Night Nurse's Reports. Bound & unbound.

### DISTRICT NURSING ASSOCIATIONS'

Case, Visit, Time, and Loan and Account Books.

### *INSTITUTIONAL LIBRARY.*

THE SCIENTIFIC PRESS will procure literature, professional and otherwise, for the formation of and addition to Institutional Libraries. It is economy of time and expenditure to utilise one source of supply.

THE SCIENTIFIC PRESS, LTD.,

28 and 29 Southampton Street, Strand, London, W.C.

